# MMTV-like Viruses and Human Breast Cancer: Evidence for Causality

**DOI:** 10.3390/cimb48020157

**Published:** 2026-01-30

**Authors:** Mónica L. Acevedo, Francisco Aguayo, Julio C. Osorio, Luis N. Ardiles, Gloria M. Calaf

**Affiliations:** 1 Laboratory of Molecular and Cellular Virology, Núcleo Interdisciplinario de Microbiología, Instituto de Ciencias Biomédicas, Facultad de Medicina, Universidad de Chile, Santiago 8330015, Chile; 2 Laboratory of Oncovirology, Departamento de Ciencias Biomédicas, Facultad de Medicina, Universidad de Tarapacá, Arica 1000000, Chile; cejulio704@gmail.com (J.C.O.); lardilesa@academicos.uta.cl (L.N.A.); 3 Instituto de Alta Investigación, Universidad de Tarapacá, Arica 1000000, Chile

**Keywords:** breast, cancer, MMTV, virus

## Abstract

Mouse Mammary Tumor Virus (MMTV) is an established mammary carcinogen in mice, yet the relevance of MMTV-like agents to human breast cancer remains debated. Across cohorts worldwide, PCR-based detection of MMTV-like DNA, in situ RNA localization, and immunohistochemical detection of viral proteins have been reported in a subset of tumors and, in some studies, in pre-invasive lesions; however, results are heterogeneous and vulnerable to methodological confounding, including murine DNA contamination and variable assay design. Here, we synthesize the evidence through a causality-oriented framework that integrates (i) standardized multi-target detection with mandatory contamination controls, (ii) epidemiologic designs that explicitly stratify sporadic versus hereditary/BRCA-driven disease, and (iii) mechanistic endpoints that are demonstrably human-relevant (e.g., in situ viral RNA/protein in tumor cells, integration-site mapping, and functional consequences of viral gene products in human models). Given current evidence, the overall causal plausibility is best considered “possible,” rising to “probable” only for a restricted subset of sporadic tumors, provided that future studies verify bona fide infection in situ using standardized multi-target assays, rigorous murine exclusion controls, and mechanistic evidence linking viral expression and/or integration to tumor cell biology. Without these endpoints, association studies alone are unlikely to resolve causality or enable meaningful clinical translation.

## 1. Introduction

Breast cancer (BC) is the most frequently diagnosed cancer in women globally, with an estimated 2.3 million new cases (11.7% of all cancers), and is the leading cause of cancer-related mortality among women, accounting for approximately 685,000 deaths in 2020 [1,2]. The heterogeneity of BC is recognized through various biomarkers with therapeutic implications. These include estrogen receptor (ER), progesterone receptor (PR) [3], human epidermal growth factor receptor 2 (HER2) [4], BC susceptibility genes 1 and 2 (*BRCA1/2*) [5], estrogen receptor 1 (*ESR1*) gene mutations, *HER2* gene mutations [6], microsatellite instability (MSI) [7], phosphatidylinositol-4,5-bisphosphate-3-kinase catalytic subunit alpha (*PIK3CA*) gene mutations [8], neurotrophic tyrosine receptor kinase (NTRK) gene fusions [9], and programmed death-ligand 1 (*PD-L1*) expression [10]. The pathogenesis of BC is complex and multifactorial, involving the interplay of reproductive hormones, environmental exposures, socioeconomic factors, smoking, stress, and dietary patterns [11,12]. Intriguingly, associations between viral infections such as Epstein–Barr virus (EBV) [13], human papillomaviruses (HPVs) [14], hepatitis C virus (HCV) [15], hepatitis B virus (HBV) [16], human cytomegalovirus (HCMV) [17], Kaposi sarcoma-associated herpesvirus (KSHV) [18], and bovine leukemia virus (BLV) [19] and BC development have been reported, though their precise roles in this malignancy require further clarification.

Notably, sequences related to the Mouse mammary Tumor virus (MMTV), the etiological agent of mammary cancer in mice, have been detected in human BC samples at variable frequencies. While some evidence suggests a potential involvement of MMTV or a related MMTV-like virus in human BC, epidemiological and experimental studies have not yet definitively clarified its role in the development of this disease. Related designations such as Human Mammary Tumor Virus (HMTV) and Human Betaretrovirus (HBRV) have been used to emphasize the possibility that a betaretrovirus adapted to humans, and not necessarily a direct murine spillover, may underlie the detected MMTV-like signals. In this review, instead of invoking a generic “controversy”, we focus on three concrete unresolved issues: (i) the distinction between true infection and laboratory contamination, (ii) the differential detection of MMTV-like sequences in sporadic versus hereditary BC, and (iii) the relative contribution of insertional versus protein-mediated mechanisms in human disease. Throughout this review, we use MMTV-like agents as an umbrella term for reported human signals (DNA/RNA/protein) that are homologous to MMTV, and we reserve MMTV for the murine virus; proposed labels such as HMTV are used in the literature but remain unstandardized. This review is organized around causality rather than cataloging MMTV biology: we condense murine virology to features that directly inform human plausibility (host range, transmission, and oncogenic mechanism), and we synthesize the human literature into a graded causal judgment. Throughout, we distinguish three recurring failure modes: murine DNA contamination, uncontrolled mixing of sporadic and hereditary BC, and over-extrapolation from murine insertional mutagenesis to humans, and we outline the minimal mechanistic evidence required to move the field from association to etiology. The central novelty of this review is an explicit, testable causality framework that links epidemiologic patterns to minimum detection/reporting standards and human-relevant mechanistic benchmarks needed to move the field from association to etiology.

## 2. MMTV Infection and Pathogenesis in Mice

In mice, exogenous MMTV is transmitted mainly via milk. After oral acquisition, the virus traverses the gut-associated lymphoid tissue, infecting antigen-presenting cells and B cells, where Sag-driven activation expands infected lymphocytes and amplifies viral load. Migrating infected lymphocytes traffic to the mammary gland, enabling infection of mammary epithelial cells; this immune-amplification step is central to efficient in vivo spread and helps explain the long pre-neoplastic latency typical of MMTV infection [20,21,22,23,24,25,26,27].

**Figure 1:**
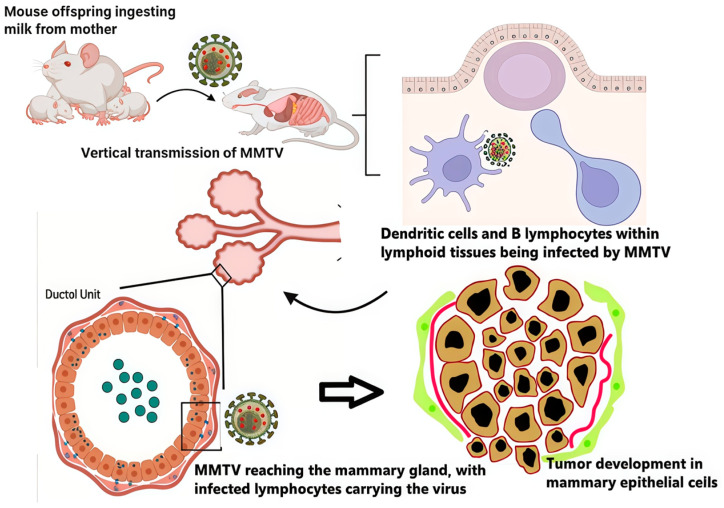
MMTV infection in the natural murine model. The vertical transmission of MMTV from mother to offspring via milk, the virus crossing the intestinal epithelium, the infection of dendritic and B cells in lymphoid tissues (GALT), the transport of the virus to the mammary gland by infected lymphocytes, and proviral integration near proto-oncogenes leading to tumor development. Proposed analogies to humans remain hypothetical and require direct in situ evidence. Created in BioRender. Osorio, J. (2026) https://BioRender.com/7ho47b0.

Murine mammary tumorigenesis is classically driven by proviral insertion near common integration sites (e.g., Wnt and Fgf family members, Notch4), where LTR enhancers dysregulate adjacent genes and tumors display clonal or oligoclonal integrations consistent with stepwise se-lection [24,25,28,29,30,31]. These features provide a clear benchmark for causality in the natural host but also highlight what is currently missing in humans: a reproducible map of tumor-cell integrations linked to transformation. Consequently, murine data should be used primarily to define testable human endpoints (integration, viral transcription/protein expression, and pathway activation) rather than as direct mechanistic proof for human BC (Figure 1).

## 3. Epidemiology of MMTV-like Sequences in Human Breast Cancer

A potential link between MMTV or related MMTV-like virus (MMTV-LV) sequences and human BC was suggested in the early 1990s [32]. Initial studies employing polymerase chain reaction (PCR) and Southern blot analysis detected sequences homologous to MMTV genes (*pol* or *env*) in BC tissues from cohorts in France (approx. 36.6%) and the USA (approx. 38.5% for specific *env* fragments). These sequences were often reported at higher frequencies in cancerous tissues compared to benign breast lesions or normal breast tissue, which prompted further interest in a possible viral contribution to human BC [33]. As additional cohorts were studied, a heterogeneous landscape emerged that can arise from genuine geographic variation in exposure and prevalence, but also from differences in methodology and cohort design, rather than from inherently “conflicting” biology [34,35,36].

Several studies, especially those originating from Australia, consistently reported relatively high detection rates of MMTV-like sequences. Ford et al. (2003) identified such sequences in 32–42% of BC cases among Australian women, a rate significantly higher than that observed in Vietnamese women residing either in Australia or Vietnam [37]. Lawson et al. (2006) detected env-like sequences in 37% of Australian BCs [38], and a later study (2018) reported the presence of MMTV p14 protein via immunohistochemistry (IHC) in 54% of cases examined [39]. Furthermore, a study by Glenn et al., also in an Australian cohort, reported a high prevalence, identifying MMTV-like sequences in 78% (39/50) of invasive BC specimens. High prevalence rates, determined using various PCR-based methodologies, were also reported in specific cohorts from Tunisia (74%) [40], Morocco (57%) [41], Pakistan (29% and 66%) [42,43], and Egypt (70–76%) [44].

Conversely, a substantial number of studies reported very low frequencies or failed to detect MMTV-like sequences altogether. For example, investigations in Austria [45], Sweden [35], and Germany (utilizing microarrays) [36] found little to no evidence of these sequences in their BC cohorts. Similarly, low prevalence rates or negative findings were frequently reported from Japan (0% in one study), Myanmar (1.7%) [46,47] and several studies conducted in Iran (e.g., 0% and 12%) [48,49]. Findings from Mexico were also contradictory, with studies reporting rates of 0% and 12.4% [50,51]. A serological study in the USA did not detect specific antibodies against MMTV proteins in BC patients [52]; given that MMTV is a milk-borne virus inducing immune tolerance in its natural murine host, and that human exposure routes and timing are unknown, negative serology does not by itself contradict a viral hypothesis. This considerable heterogeneity in reported prevalence rates is likely attributable to several factors, prominently including the wide array of detection methods employed across studies. Techniques such as standard PCR, nested PCR, quantitative real-time PCR (qPCR), in situ hybridization (ISH), IHC, and Southern blotting possess differing sensitivities and specificities, potentially influencing results [53]. Variations in the choice of target viral gene (*env*, *pol*, LTR), primer sequences, and laboratory protocols may also contribute significantly to the discordant findings. In addition, work on XMRV and related murine retroviruses clearly demonstrated that murine DNA contamination in PCR master mixes, commercial reagents, and extraction columns can generate spurious murine retroviral signals in human samples, and dedicated assays for murine mitochondrial DNA or IAP sequences are therefore essential to exclude contamination in MMTV-like studies [54,55,56,57,58]. Taken together, the striking geographic heterogeneity is unlikely to have a single explanation; plausible contributors include differences in sample types (fresh vs. FFPE), nucleic-acid integrity, target loci/primer sets, assay sensitivity (PCR vs. qPCR/ddPCR), laboratory workflows, and contamination control rigor, as well as true population-level effects such as host genetic background, reproductive/hormonal factors, environmental co-exposures, and circulating mouse reservoir ecology. Publication bias and selective reporting of positive findings should also be considered, particularly where methods and negative controls are incompletely described. Across cohorts, apparent prevalence is strongly assay dependent. Conventional PCR and nested PCR maximize analytic sensitivity but are the most vulnerable to amplicon carryover and low-level murine DNA contamination; qPCR improves quantification but remains target- and primer-dependent; ddPCR can increase precision at low copy number but does not solve contamination or locus-specificity by itself [59]. In situ assays (e.g., ISH/RNAscope for RNA; IHC for protein) provide cellular localization and reduce the key confounder of detecting exogenous DNA not residing in tumor cells but typically trade sensitivity for specificity and are constrained by probe/antibody validation [60]. NGS-based approaches can, in principle, resolve integration or clonality, yet require stringent negative controls, laboratory separation, and transparent reporting of read-level filters to avoid artefactual signals [61]. Beyond simple cohort stratification, the biology of BRCA1/2-associated tumors could plausibly modulate detectability or relevance of any MMTV-like signal. For example, BRCA1-driven cancers are enriched for basal-like/triple-negative phenotypes and exhibit profound genomic instability; these features could either (i) reduce selective advantage for a virus that acts through hormone-responsive or luminal programs, or (ii) complicate downstream interpretations by increasing background DNA damage and rearrangements. Conversely, if any subset of sporadic tumors harbors bona fide viral material in tumor cells, that subset should be defined by tumor-intrinsic evidence (multi-locus DNA plus in situ RNA/protein), not by geography alone. Importantly, these are hypotheses for future studies.

MMTV-like sequences or related proteins have been detected not only in invasive BCs but also in other contexts, including normal breast epithelium adjacent to tumors, ductal carcinoma in situ (DCIS), atypical ductal hyperplasia (ADH), and benign breast lesions [62]. Rather than “complicating” interpretation, higher positivity rates in pre-invasive lesions and detection in adjacent epithelium are compatible with models in which infection is widespread and longstanding, and in which early viral contributions may be followed by “hit-and-run” or selection events during progression [63,64,65]. An association with BC subtypes has also been proposed. A particularly informative study by Naccarato et al. reported that MMTV-like sequences were significantly associated with sporadic BC (30.3% prevalence) but were almost absent in hereditary BC (4.2% prevalence). This suggests that cohort heterogeneity, specifically the uncontrolled inclusion of HBC cases with known genetic etiologies (e.g., *BRCA1/2* mutations), may be a primary confounding variable that accounts for some of the negative findings reported in the literature [66]. These results underscore the importance of distinguishing sporadic and hereditary disease in both individual studies and meta-analyses, and they support the notion that a virus, if present, may preferentially contribute to sporadic BC rather than to BRCA-driven tumors.

A meta-analysis published in 2014 attempted to synthesize data from available studies up to that point. It reported a significantly higher overall estimated prevalence of MMTV-like sequences in Western populations compared to Asian populations and calculated a significant summary odds ratio (OR = 15.20) for the association with BC risk, based on the included studies [67]. However, the authors acknowledged the substantial heterogeneity among the studies, which remains a major limitation in drawing firm conclusions from pooled data. Taken together, the epidemiological evidence reveals a recurrent, geographically patterned signal of MMTV-like detection in BC that is strongest in certain Western and Middle Eastern cohorts, but its magnitude and meaning remain uncertain without harmonized detection methods, rigorous contamination controls and explicit stratification of sporadic versus hereditary disease (Table 1).

## 4. Mechanisms of MMTV-Mediated Carcinogenesis in Humans

**Figure 2:**
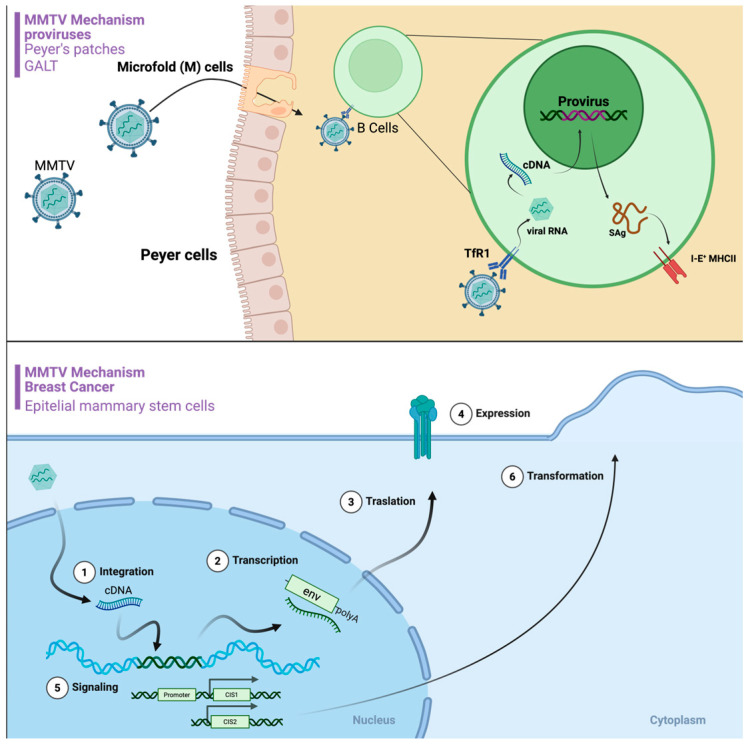
Proposed mechanism of MMTV provirus entry into B cells (**Top**) and MMTV-induced breast cancer in epithelial cells (**Bottom**). (**Top**) MMTV proviruses enter the host through M (Microfold) cells in Peyer’s patches. MMTV is then transferred to B cells, which express the transferrin receptor 1 (TfR1), allowing the virus to enter the cell (in mice). Inside the B cell, MMTV’s viral RNA is reverse transcribed into cDNA, which integrates into the host’s genome as a provirus. The provirus expresses viral RNA and a superantigen (SAg). The SAg binds to MHC II, stimulating B-cell proliferation. (**Bottom**) The MMTV provirus can also infect epithelial cells. (1) The MMTV cDNA integrates into the host cell’s genome, often near regulatory genes such as CIS1 or CIS2. (2) The integrated provirus undergoes transcription, producing viral mRNA with a poly-A tail. (3) The viral mRNA is translated into viral proteins, such as env. (4) The viral proteins are expressed on the cell surface. (5) The integration of the provirus can also lead to signaling changes within the cell, particularly due to the proximity of the provirus to the CIS1 and CIS2 genes. (6) These combined effects lead to the transformation of the epithelial cell, which can result in BC. Created in BioRender. Gangadaran, P. (2025). https://BioRender.com/g8k2s3b.

While insertional mutagenesis is the established oncogenic mechanism in mice, evidence for an analogous dominant mechanism in humans remains limited. No reproducible map of common integration sites in human BC tissues is currently available, and the presence of MMTV-like sequences in human samples has not yet been linked to recurrent insertional activation of specific proto-oncogenes [79]. Instead, research has focused on non-insertional mechanisms driven by specific MMTV proteins. Of particular interest is the MMTV envelope protein, which has been proposed to potentially facilitate the transformation of normal human breast cells. This protein contains a distinctive immunoreceptor tyrosine-based activation motif (ITAM), a motif typically associated with immune cell signaling [80]. When the MMTV Env protein is experimentally expressed in human breast epithelial cells, either in vitro cultures or through genetic manipulation in mice, its functional ITAM appears to disrupt normal acinar morphogenesis and induce hallmarks of transformation, including anchorage-independent growth and invasion [81]. This disruption is associated with the activation of oncogenic signaling pathways, including Syk and Src tyrosine kinases, that promote hallmarks of cancer, such as aberrant proliferation, invasion of surrounding tissues, and resistance to apoptosis [82]. Crucially, mutations that inactivate the ITAM function (e.g., Y > F substitutions) eliminate these transforming effects in experimental systems [83]. This proposed mechanism is significant as it does not rely on insertional mutagenesis near specific proto-oncogenes, pointing to a potential mechanism by which a viral protein could theoretically contribute to the development of human BC [82] (Figure 2). Importantly, most mechanistic evidence arises from murine tumors, murine cell systems, or engineered expression models; these studies establish biological plausibility but should not be overinterpreted as proof of active viral replication or an etiologic role in human breast carcinogenesis without in situ validation in human tissues.

The MMTV Env signal peptide protein, p14, despite being a fragment of the larger envelope precursor, appears to possess unique functionalities within host cells. P14 is a small protein consisting of 98 amino acids and plays a role in transporting the Env precursor protein (74 kDa) produced by MMTV through the endoplasmic reticulum (ER). Initially designated as MMTV-p14, it is commonly referred to simply as p14 [84]. Once inside the ER, a signal tag is removed, allowing for post-translationally modifications involving sugar addition, leading to the maturation of viral envelope proteins gp52 and gp36 [85]. Typically, signal peptides are degraded by enzymes after directing proteins to the ER, but the p14 signal peptide appears to be an exception [86]. Research has indicated that it can localize to the nucleoli in the cells of mice with mammary tumors and lymphoma induced by the virus, as well as potentially in human BC, suggesting that the p14 signal peptide can shuttle between the nucleus and cytoplasm [87]. Notably, the phosphorylation state of p14 appears to modulate the tumorigenic potential of MMTV-infected cells in mouse models. The envelope precursor signal peptide functions as a potential tumor-modulating phosphoprotein, undergoing phosphorylation by two serine kinases: casein kinase 2 (CK2) at serine 65 and protein kinase C (PKC) at serine 18 in experimental settings [86]. Studies using site-directed mutagenesis found that a p14-Ser65Ala mutation was associated with impaired tumorigenicity, whereas a p14-Ser18Ala mutation was associated with enhanced tumorigenicity. This suggests that phosphorylation at serine 65 (by CK2) is pro-oncogenic, while phosphorylation at serine 18 (by PKC) is anti-oncogenic. Thus, the tumorigenic potential of MMTV in these models may be linked to CK2 and PKC activity on p14. Excessive amounts of p14 might accelerate tumor growth. p14 exhibits a range of functions, as it can be in both the nucleolus and the ribosome. Furthermore, p14 may act as a regulatory switch, potentially affecting the localization of crucial RNA components relative to the nucleolus and impacting ribosome assembly in the cytoplasm. This process could lead to increased protein production and disturbances in cell growth [88]. Moreover, microarray analysis associated with these phosphorylation states appears to determine its functional role in these models. This analysis has been associated with the transcriptional regulation of genes related to cell proliferation and the Erb-B signaling pathway, which is well-known for its role in driving oncogenesis [89]. Overall, the overexpression of p14 can disrupt cellular homeostasis by affecting protein synthesis and potentially activating pro-cancerous signaling pathways, underscoring its potential role as an oncogenic factor in experimental contexts.

The p14 protein is not only a signaling molecule but also functions as part of a variant of MMTV-env, known as Rem (Regulator of Export of MMTV mRNA), which facilitates the transport of specific viral mRNAs out of the nucleus [90,91]. This finding adds to the complexity of MMTV. Additionally, p14 can be present on the surface of cells infected with MMTV, presenting a potential target for developing therapies aimed at MMTV-related cancers [92]. A study by Braitbard et al. in 2016 explored the potential of p14 for treating MMTV-associated tumors in mice [91]. Their results indicate several promising avenues for further exploration. p14, or modified versions of it, could potentially be used in vaccines or antibody therapies. Furthermore, immune cells specifically targeting p14 on cancer cells were identified as viable treatment options in their murine model. The study also suggests the possibility of introducing molecules or inhibitors directly into cancer cells to target p14. Finally, both p14 and antibodies designed against it could potentially be used for early detection of these tumors. Although the detection of p14 in MMTV-positive human cases has been reported, further research is necessary to substantiate these therapeutic possibilities [92].

MMTV encodes various proteins beyond the essential structural and enzymatic components typically found in retroviruses. These regulatory proteins, such as Rem, Sag, and Naf, play crucial roles in the viral life cycle, influencing critical processes such as RNA splicing, nuclear export, and immune evasion [93]. Suggestions that additional, as-yet-undiscovered MMTV proteins might have Tax-like oncogenic properties in humans remain speculative and are not supported by current evidence; we therefore avoid extending mechanistic models beyond what is directly demonstrated [94]. Intriguingly, some of these MMTV proteins have been linked to BC development in mice. For example, Rem, which is involved in RNA export, could potentially impact the expression of cellular genes related to cell proliferation and survival. Likewise, Sag, a superantigen, has the potential to disrupt the immune response, possibly fostering an environment favorable for tumor growth [95]. In addition to direct viral-driven mechanisms, the interplay between the virus and host innate immunity is also being investigated. The APOBEC3 (Apolipoprotein B mRNA Editing Enzyme Catalytic Polypeptide-like 3) family of cytidine deaminases are key antiviral restriction factors that can induce G-to-A hypermutations in retroviral cDNA during reverse transcription. While this is a host defense mechanism, *APOBEC* mutagenesis is also a known “off-target” source of somatic mutations in the host genome, creating a distinct mutational signature observed in many human cancers. A potential link was observed in human BC, where MMTV-like positivity combined with an *APOBEC3A/B* gene deletion was associated with a significantly earlier age-at-diagnosis. This suggests a complex interplay where the virus may trigger a host mutagenic response that, in turn, contributes to carcinogenesis, or where a compromised innate immune defense (i.e., *APOBEC3A/B* deletion) enhances the pathogenic potential of the virus [96]. These findings are consistent with a model in which viral infection, insertional events and host restriction mechanisms converge to shape tumor evolution, but they do not yet define a single dominant pathway in human BC. Overall, these pathways support plausibility but are not yet anchored to definitive viral expression or integration in human tumor cells, underscoring the need for paired in situ detection and functional validation in primary-like human systems. Figure 2 is a conceptual model (hypothesis-generating) largely derived from murine retrovirology; the proposed steps and arrows should not be interpreted as established mechanisms in human breast cancer and require direct in situ and integration-level validation in human tissues.

## 5. Human and MMTV Interactions

If MMTV-like signals in human tissues reflect true infection, two non-exclusive scenarios require testing: recurrent zoonotic exposure from mice (potentially modulated by the geographic distribution of Mus musculus domesticus) and/or sustained circulation of a human-adapted betaretrovirus derived from an ancient cross-species transmission event [97,98,99,100,101]. Putative routes such as saliva or other mucosal shedding remain intriguing but technically vulnerable to contamination artifacts; therefore, progress depends on orthogonal detection in paired tissues, deep sequencing with stringent murine DNA exclusion, and phylogenetic evidence of viral evolution inconsistent with laboratory mouse proviruses [54,55,56,57,58].

## 6. Evidence Synthesis, Causal Plausibility and Perspectives

MMTV is unequivocally oncogenic in the murine mammary gland, but in humans the cumulative literature remains insufficient for a definitive etiological claim. A central limitation is that epidemiological heterogeneity (including true geographic variation) is inseparable from methodological variability and the long-recognized risk of murine DNA contamination; therefore, additional prevalence surveys alone are unlikely to resolve causality without mechanistic, human-relevant endpoints (Table 2).

Operationally, we define the ‘restricted subset’ as sporadic tumors that meet a minimum evidentiary threshold: (1) concordant detection of at least two independent viral loci (e.g., env and LTR/pol) in the same specimen with rigorous murine-contamination controls; (2) localization of viral RNA and/or protein within tumor epithelial cells by validated in situ methods; and (3) ideally, integration or clonality evidence by orthogonal sequencing-based approaches. Tumors not meeting these criteria should be interpreted as unconfirmed signals. We therefore position the overall causal relationship between MMTV-like agents and human breast cancer as “possible”, rising to “probable” only in a restricted subset of sporadic tumors, provided that future studies demonstrate bona fide infection in situ with standardized multi-target assays, rigorous murine contamination exclusion, and mechanistic evidence linking viral expression and/or integration to tumor cell biology. This grading is intentionally conservative: the current literature is limited by interlaboratory heterogeneity, incomplete reporting of negative controls, and the risk that strong non-viral etiologies (e.g., hereditary/*BRCA*-driven disease) dilute or mask any viral signal when cohorts are not stratified.

A key implication of this grading is methodological: progress requires a unified detection and reporting framework and a shift toward human-relevant causal benchmarks. At minimum, future studies should report and/or implement (i) multi-locus nucleic-acid detection (e.g., LTR + *env* + *gag/pol*) with quantitative readouts (qPCR/ddPCR) and sequence confirmation; (ii) mandatory murine exclusion controls (IAP and/or mitochondrial markers), physical separation of pre-/post-PCR steps, and reagent/environmental blanks; (iii) orthogonal localization in tissue (RNAscope/ISH for viral RNA and IHC for viral proteins) with clear tumor-cell attribution; (iv) cohort design with explicit stratification (sporadic vs. hereditary/*BRCA*, tumor subtype, grade/stage, and relevant exposures) and preferably prospective sampling; (v) where signals are detected, integration-site mapping (NGS-based) and assessment of clonality/single-cell distribution; and (vi) functional testing in primary-like human models (organoids, patient-derived models) to connect viral gene products to defined oncogenic phenotypes. Clinically, translation is premature, but if a causal subset is validated, it could enable risk stratification, standardized biomarker development, and rational exploration of prevention/therapeutic strategies (e.g., vaccination concepts or antiviral/immune-targeted approaches) tailored to that subset.

## Figures and Tables

**Table 1:** Prevalence of MMTV-like Sequences/Proteins in Human Breast Tissues Worldwide (Selected Studies).

First Author	Year	Country	Method	Positive/Total	Prevalence (%)	Ref.
Moyret C	1992	France	PCR, SB	NA (Not reported)	NA	[32]
Wang Y	1995	USA	PCR	121/314	38.5	[33]
Melana SM	2002	USA (Arg.)	PCR	23/74	31.0	[68]
Ford CE	2003	Australia/ Vietnam	PCR	A1: 19/45; A2: 1/120; V: NR (reported as % only)	A1: 42.2; A2: 0.8; V: 1.8	[37]
Witt A	2003	Austria	PCR	0/50	0.0	[45]
Mant C	2004	UK	PCR	7/44	16.0	[34]
Ford CE	2004	Australia	PCR	43/136	32.0	[69]
Levine PH	2004	USA(Tunisia)	PCR	29/39	74.0	[40]
Goedert JJ	2006	USA	IB, IP	0/92	0.0	[52]
Luo T	2006	China	PCR	22/131	16.8	[70]
Lawson JS	2006	Australia	PCR, SB	22/59	37.3	[38]
Zapata-Benavides P	2007	Mexico	PCR	5/119	4.2	[71]
Bindra A	2007	Sweden	qPCR	0/18	0.0	[35]
Frank O	2008	Germany	MA	0/23	0.0	[36]
Fukuoka H	2008	Japan	PCR, SB	0/46	0.0	[46]
Hachana M	2008	Tunisia	PCR	17/122	13.9	[72]
Mazzanti CM	2011	Italy	PCR, qPCR, ISH	ADH: 6/22; DCIS: 40/49; IDC: 7/20; Normal tissue: 6/32	ADH: 27.0; DCIS: 82.0; IDC: 35.0; Normal Tissue: 19.0	[62]
Motamedifar M	2012	Iran	PCR	0/300	0.0	[48]
Glenn WK	2012	Australia	PCR	39/50	78.0	[73]
Morales-Sánchez A	2013	Mexico	PCR	0/86	0.0	[50]
Slaoui M	2014	Morocco	PCR, Seq	24/42	57.1	[41]
Cedro-Tanda A	2014	Mexico	PCR, Seq.	57/458	12.4	[51]
Reza MA	2015	Iran	PCR, IHC	12/100	12.0	[49]
San TH	2017	Myanmar	PCR, Seq.	1/58	1.7	[47]
Lawson JS	2017	Australia	PCR	9/25	36.0	[65]
Naushad W	2017	Pakistan	PCR	73/250	29.3	[42]
Shariatpanahi S	2017	Iran	PCR	19/59	32.2	[74]
Lawson JS	2018	Australia	IHC/PCR	IHC: 27/50; PCR: 12/45	IHC: 54.0; PCR: 27.0	[39]
Al Dossary R	2018	Saudi Arabia	PCR, Seq.	6/103	5.9	[63]
Seo I	2019	South Korea	PCR	12/128	9.4	[75]
Naccarato AG	2019	Italy	PCR, IHC	HBC: 2/47; SBC: 17/56	HBC: 4.2; SBC: 30.3	[66]
Al Hamad M	2020	Italy (Jordan)	qPCR, ISH	11/100	11.0	[76]
de Sousa Pereira N	2020	Brazil	PCR, Sequencing	BC: 41/217 Blood: 17/32	BC: 19.0; Blood: 53.0	[64]
Loutfy SA	2021	Egypt	PCR, Sequencing	HBC: 21/30 SBC: 38/50	HBC: 70.0; SBC: 76.0	[44]
Wang FL	2021	China	Nested PCR	21/119	17.6	[77]
Khalid HF	2021	Pakistan	qPCR	69/105	65.7	[43]
Gupta I	2021	Qatar	PCR	5/70	7.0	[78]

Abbreviations: BC, Breast Cancer; IDC, Invasive Ductal Carcinoma; DCIS, Ductal Carcinoma In Situ; ADH, Atypical Ductal Hyperplasia; SBC, Sporadic Breast Cancer; HBC, Hereditary Breast Cancer; PCR, Polymerase Chain Reaction; qPCR, Quantitative Real-Time PCR; ISH, In Situ Hybridization; IHC, Immunohistochemistry; SB: Southern blotting; Seq: Sequencing; MA: Microarrays.

**Table 2:** Evidence synthesis for MMTV-like sequences in human breast cancer: methodological requirements.

Designs and Assays for a Causal Inference	What the Literature Currently Shows	Evidence Showed
Standardized multi-target assays (LTR/*env*/*pol*) plus RNA/protein localization in the same tumor cells, with pre-registered murine contamination controls.	Highly variable MMTV-like detection across cohorts; enrichment in DCIS/ADH or adjacent epithelium is reported in some studies; negative studies also exist [32,33,34,35,36,37,38,39,40,41,42,43,44,45,46,47,48,49,50,51,52,53,54,55,56,57,58,67].	Detection in tumors (DNA/RNA/protein)
Prospective designs with explicit stratification (sporadic vs. hereditary/BRCA1/2) and harmonized pathology and molecular subtyping.	Signals appear stronger in sporadic BC in at least one cohort and may be diluted by inclusion of hereditary/*BRCA*-driven tumors [62,63,64,65,66,67].	Sporadic vs. hereditary disease
Integration-site mapping at clonal or single-cell resolution, coupled to viral transcription/protein expression and cellular pathway readouts.	No reproducible map of common integration sites or clonal proviral structure has been established in human BC [24,28,29,79].	Replication and integration benchmarks
Demonstration that proteins are expressed in human tumor cells in situ and that downstream signaling correlates with viral positivity and functional phenotypes.	Env ITAM and p14 can reprogram signaling and cellular homeostasis in experimental systems, providing testable non-insertional mechanisms [80,81,82,83,84,85,86,87,88,89,90,91,92,93,94,95].	Mechanistic plausibility
Phylogenetic evidence of viral evolution in humans, and reproducible detection in reservoirs (e.g., saliva) using orthogonal assays with stringent contamination exclusion.	Zoonotic and human-adapted scenarios remain hypotheses; evidence is suggestive but not decisive [54,55,56,57,58,97,98,99,100,101].	Transmission/origin

## Data Availability

No new data were created or analyzed in this study. Data sharing is not applicable to this article.
